# Conversion of Fructose to HMF in a Continuous Fixed Bed Reactor with Outstanding Selectivity

**DOI:** 10.3390/molecules23071802

**Published:** 2018-07-20

**Authors:** Eric Weingart, Sarah Tschirner, Linda Teevs, Ulf Prüße

**Affiliations:** Thuenen Institute of Agricultural Technology, Bundesallee 47, 38116 Braunschweig, Germany; eric.weingart@thuenen.de (E.W.); sarah.tschirner@thuenen.de (S.T.); linda.teevs@thuenen.de (L.T.)

**Keywords:** HMF, continuous fixed bed reactor, heterogeneous catalysis, HFIP, fructose

## Abstract

5-Hydroxymethylfurfural (HMF) is a very promising component for bio-based plastics. Efficient synthesis of HMF from biomass is still challenging because of fast degradation of HMF to by-products under formation conditions. Therefore, different studies, conducted mainly in monophasic and biphasic batch systems with and without water addition have been published and are still under investigation. However, to produce HMF at a large scale, a continuous process is preferable. Until now, only a few studies have been published in this context. In this work, it is shown that fluorous alcohol hexafluoroisopropanol (HFIP) can act as superior reaction solvent for HMF synthesis from fructose in a fixed bed reactor. Very high yields of 76% HMF can be achieved in this system under optimized conditions, whilst the catalyst is very stable over several days. Such high yields are only described elsewhere with high boiling reaction solvents like dimethylsulfoxide (DMSO), whereas HFIP with a boiling point of 58 °C is very easy to separate from HMF.

## 1. Introduction

Renewable resources are promising alternatives to dwindling fossil resources for the production of important platform chemicals. One of the most interesting organic compounds that can be obtained from renewable raw materials is 5-hydroxymethylfurfural (HMF). Because of its versatile functionality, HMF is an important key intermediate in the production of various bio-based chemicals.

A promising HMF oxidation product is furandicarboxylic acid (FDCA). FDCA is the major building block for polyethylene furanoate (PEF), a bio-based alternative for the important plastic polyethylene terephthalate (PET). The market volume of PET of approx. 50 million t/a illustrates the potential and the importance of HMF. Different monophasic reaction systems like water, DMSO, ionic liquids or biphasic reaction systems in batch mode have been applied for the dehydration of fructose to produce HMF. Promising results with HMF yields and selectivities up to 90% are achieved using organic solvents or biphasic reaction systems [1,2,3,4]. However, in order to produce sufficient quantities of HMF, a continuous production is required. Until today, only a few studies in the field of continuous single phase as well as continuous biphasic systems for HMF production have been carried out. Tuercke et al. [5] used a continuous microreaction process for HCl catalysed dehydration of aqueous fructose. In this single phase, a fructose conversion of 71% with an HMF yield of 54% and a selectivity of 75% could be achieved. The addition of the co-solvent DMSO led to an increase in HMF selectivity to 85%. Jeong et al. [6] used continuously generated HMF as an intermediate to produce different heterocyclic furan chemicals. They used a functionalized capillary micro reactor for catalytic dehydration of fructose in DMSO. At a reaction temperature of 150 °C and a reaction time of 6 min, both the conversion and selectivity reached 99%. Schön et al. [7] also used a monophasic continuous system for HMF production. With fructose in DMSO as a starting material and quartz sand-based cartridge as a reactor, a reaction temperature of 180 °C and a flow rate of 0.6 mL/min, HMF yields of 90.3% could be achieved. However, the use of DMSO as a solvent is not preferable. Because of its high boiling point (189 °C), the products cannot easily be separated, and DMSO degrades at higher temperatures in acidic reaction systems [8].

A few studies can also be found on the use of biphasic reaction systems for the continuous production of HMF. The biphasic solvent system of a 0.25 M aqueous solution of fructose and methylisobutylketone (MIBK) as the extraction phase led to an HMF yield of 74% [9]. Using similar systems, Shimanouchi et al. [10] and Lueckgen et al. [11] achieved 88% yield and 93% yield, respectively, in the MIBK phase with a fructose solution of 10 wt%. In another system, Muranaka et al. [12] described the formation of HMF in a micro reactor with a yield of 80.9%, using phosphate buffer saline as the reaction phase and 2-*sec*-butyl phenol (2BP) as the extraction phase.

A disadvantage of all the mentioned research is the use of solvents with high boiling points and the resulting difficult separation of the thermally unstable HMF. Furthermore, the yield of HMF in biphasic systems is the sum of HMF in aqueous and organic phases, which leads to the problem of separating HMF from the catalysts in aqueous phase.

**Scheme 1:**
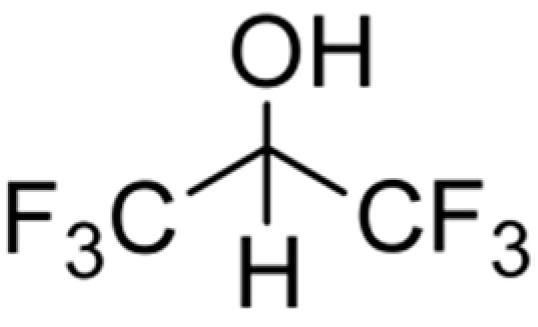
HFIP structure.

In our previous work [13,14], we showed in a batch system that hexafluoroisopropanol (HFIP, Scheme 1) in combination with water and with the use of an acidic ion exchanger (Lewatit K2420 or Amberlyst 15) is a very well-suited monophasic system for the dehydration of fructose to HMF.

HFIP is characterised by a low boiling point of 58 °C, which leads to low thermal stress for HMF and makes it easy to separate the solvent. In a batch reaction system, we achieved an HMF yield of 76% at complete conversion of fructose [14]. Therefore, we want to enter the next stage for an industrial process by testing HFIP as a solvent in a continuous fixed bed reactor.

## 2. Results and Discussion

**Figure 1:**
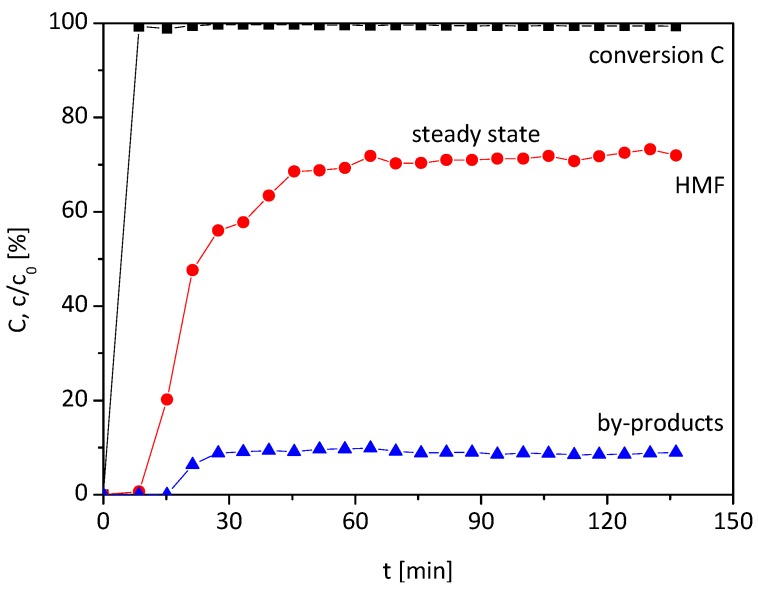
Reaction course in a continuous fixed bed reactor until a steady state was reached (0.1 M fructose, 105 °C, 15 vol% H_2_O in HFIP, residence time τ = 20 min, 1.7 g Lewatit K2420 catalyst bed).

As already mentioned, our preliminary study [14] of the synthesis of HMF from fructose in HFIP with the heterogeneous catalyst Lewatit K2420 in a batch reactor showed a high yield. As consequence of this study, a fixed bed reactor based on Lewatit K2420 as a catalyst bed was designed (see Figure 6 in Section 3.1). A fructose/HFIP/water solution was pumped through the reactor and samples were taken, which resulted in the following reaction course (Figure 1).

In the first sample, after seven minutes, fructose, HMF or the by-products formic acid and levulinic acid are detectable, because the complete feed is adsorbed in the catalyst bed. With increasing time, HMF and the by-products are detected, whilst the fructose concentration is zero. Around 80% of fructose is converted into HMF and the by-products formic and levulinic acid. The remaining 20% fructose is expected to be converted into undetectable humins. After a certain time, the HMF and by-product concentrations do not change significantly. Therefore, a steady state is reached, whereby the amount of time for the different compound concentrations to reach the steady state differs. This means that HMF and the by-products interact with the catalyst bed differently, which may be explained by the fact that the acidic by-products and the acidic catalyst bed will repel each other, whereas the neutral HMF will not.

Knowing this behavior, the following different experiments were performed. To ensure reliable results for each change of reaction temperature (95–105 °C) and H_2_O content in HFIP (12.5–17.5 vol% H_2_O in HFIP), the average of three samples was calculated, which were taken after a minimum of the triple residence time τ. In all experiments, standard deviations of <1% for conversion, yield or by-product formation were calculated; therefore, no error bars are shown in the figures. The chosen reaction conditions are based on the optimized conditions of the prior study [14] in a batch reaction system.

**Figure 2:**
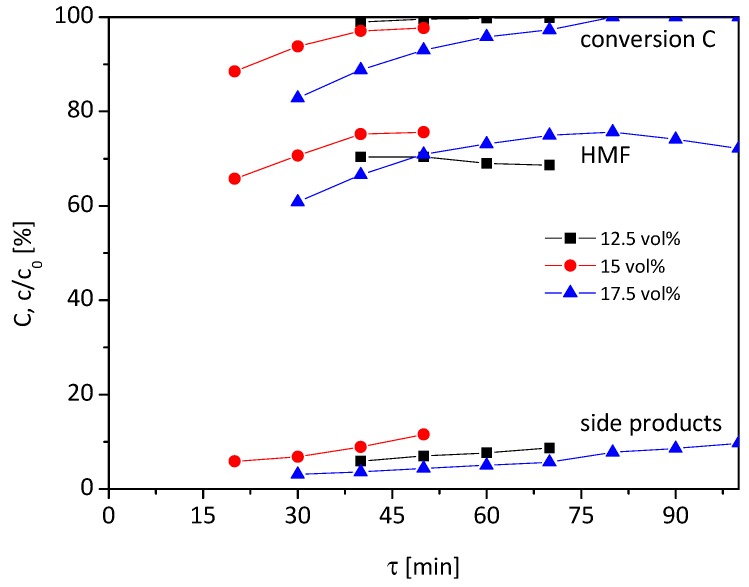
Influence of water content on conversion, HMF yield and by-products (0.1 M fructose, 95 °C, 12.5–17.5 vol% H_2_O in HFIP, 1.7 g Lewatit K2420 catalyst bed).

At first, the temperature of the catalyst bed was adjusted to 95 °C, and the water content of the reaction solution was varied in the range of 12.5–17.5 vol% (Figure 2).

At a water content of 12.5 vol% (black squares), the complete conversion of fructose is observed for all tested τ higher than 40 min. The course of the graph of HMF is the reverse. For τ = 40 min the highest HMF yield of 70% is observed, which drops slightly to 68% at higher τ. Also, the by-products are formed at higher rates from 5 to 11% with rising residence time. In sum, the course of the graphs are only slightly influenced by residence time at a water content of 12.5 vol%. If the water content is raised to 15 or 17.5 vol%, the influence of the residence time on conversion and yield increases. At 15 vol% (red circles), the HMF yield enhances gradually from 66 to 75% and conversion from 88 to 97% when the residence time is extended from 20 to 40 min. Further extension of the residence time to 50 min has no effect on the HMF yield, only the conversion and by-product formation increase. However, the variation of τ has no effect on the selectivity which remains around 75%.

For the 17.5 vol% water content (blue triangles), the course of the graph is similar to 15 vol%. At the low residence time of 30 min, a conversion rate of 83% is reached, with 61% HMF yield and 3% yield of by-products. When extending τ to 80 min, a full conversion and maximum HMF yield of 76% are achieved, whilst 8% by-products are formed. With further prolonging of τ, the yield of HMF drops again and by-product formation increases, which lowers the selectivity.

So all in all, the highest HMF yield of around 75% is achieved with a water content of 15 or 17.5 vol% and when the residence time is adjusted accordingly. Below 15 vol% water content, only 70% HMF yield can be achieved at 95 °C. The selectivity does not change significantly by variation of residence time when fructose is not completely converted for all three tested water contents.

**Figure 3:**
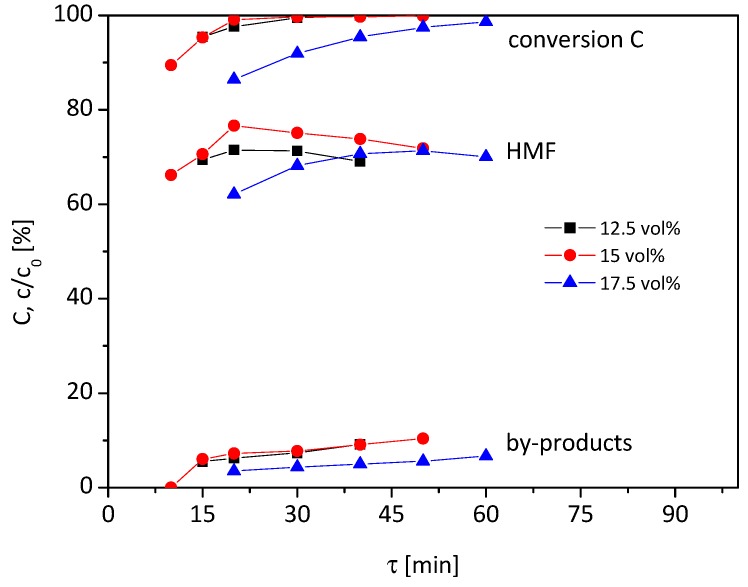
Influence of water content on conversion, HMF yield and by-products (0.1 M fructose, 100 °C, 12.5–17.5 vol% H_2_O in HFIP, 1.7 g Lewatit K2420 catalyst bed).

In Figure 3, the graphs of the reaction solutions with a 12.5–17.5 vol% water content at a higher reaction temperature of 100 °C are shown. Due to the higher temperature, shorter residence times were chosen compared to the reaction at 95 °C (Figure 2).

For the 12.5 vol% water content, the graphs at 100 °C show similarities to the graphs at 95 °C. The HMF yield rises with increasing residence time to approx. 70% at 20 min with a high conversion rate and decreases again when the residence time exceeds 20 min. However, the graphs are only slightly affected by residence time. If the water content is increased to 15 vol%, the graphs have a similar course in the case of conversion and by-product formation, but differ in HMF yield. Compared to the graph at 12.5 vol%, a higher maximum in HMF yield of 76% can be achieved at 20 min residence time. At a lower or higher residence time, the HMF yield drops again.

When the water content is 17.5 vol%, an HMF yield of 62% at 86% conversion and 20 min residence time is reached. With rising residence time, the HMF yield increases to 71% at 50 min residence time with 97% conversion. The selectivity does not change in this period significantly and remains at 72–73%. A further increase of τ reduces the yield of HMF again.

**Figure 4:**
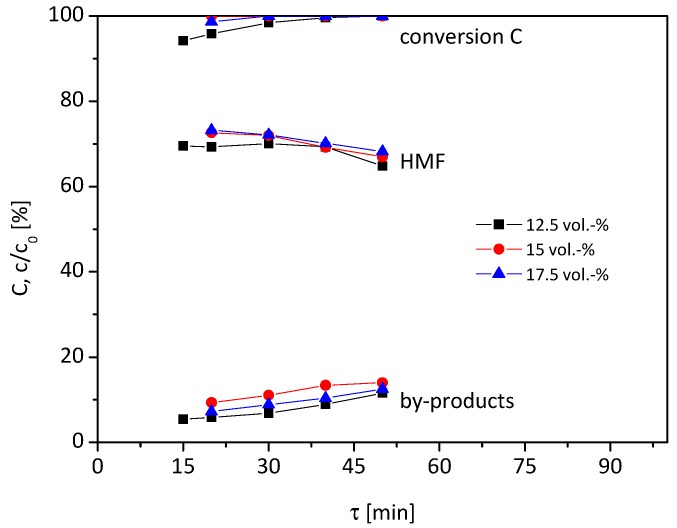
Influence of water content on conversion, HMF yield and by-products (0.1 M fructose, 105 °C, 12.5–17.5 vol% H_2_O in HFIP, 1.7 g Lewatit K2420 catalyst bed).

As a last step, the temperature was increased to 105 °C (Figure 4). Unlike the reaction at 95 °C and 100 °C, the conversion, yield and by-product formation only slightly changed by varying the residence time. The highest HMF yield of 73% is achieved with 15 and 17.5 vol% water content at 20 min residence time.

In sum, the maximum yield of HMF ranges between 70–76% for all tested water concentrations (12.5–17.5 vol%) and reaction temperatures (95–105 °C). Those belong to the highest yields of HMF reported in heterogeneous continuous reaction systems without using high boiling solvents or harsh reaction conditions (T > 110 °C, mineral acids) [1]. The highest HMF yield of 76% is achieved when using 17.5 vol% water content at 95 °C and τ = 80 min (0.162 mL/min) or 15 vol% water content at 100 °C and τ = 20 min (0.588 mL/min). These result in a space time yield of 7.6 × 10^−5^ mol/(L min), which is better than in other tested continuous processes [15], even with hard-to-separate DMSO or ionic liquids.

If the reaction is carried out under incomplete conversion of fructose, the selectivity of the process is not influenced by residence time in the investigated range. Further research has to be done to determine the reaction kinetics in order to explain this behaviour. The balance of all experiments cannot be completely explained by HPLC analysis. Therefore, the non-detected amount of 10–20% is expected to be humins, that are dissolved in the reaction solution and/or adsorbed at the catalyst surface.

**Figure 5:**
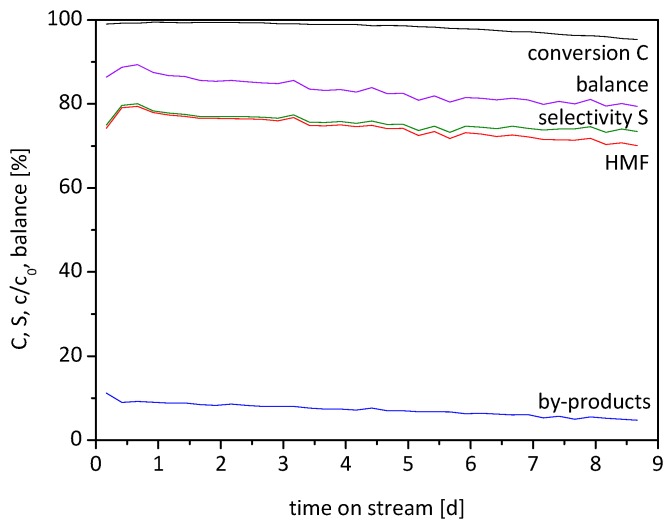
Long-term stability of the fixed bed (τ = 80 min (0.162 mL/min), 0.1 M Fructose, 95 °C, 17.5 vol% H_2_O in HFIP, 1.7 g Lewatit K2420 catalyst bed).

In the following experiment, the catalyst long-term stability over several days was evaluated. The best reaction conditions based on prior experiments were chosen: 95 °C and 17.5 vol% water content at a residence time of 80 min (Figure 5). For this experiment, three samples every six hours were taken (standard derivation of <1% for conversion, yield and by-products).

During the first hours, the yield of HMF increases from 74 to 80% at nearly complete conversion. After 8.7 days, the yield and conversion decrease gradually over time to 70% and 95%, respectively. During this time the selectivity of the HMF formation drops from 80 to 74%, which implies that the selectivity is less influenced by catalyst deactivation than the yield of HMF. The integration of the HMF curve equals an overall yield of 26.6 g or 74% HMF in those 8.7 days. The balance drops over time as well, which may be explained by adsorbed humins on the catalyst surface, which can react with HMF because of reactive functional groups. It is also conceivable that the catalyst pores become clogged by adsorbed humins, which worsens the active site accessibility and diffusion in and out of the pores. Also, leaching of acid sites is possible.

Because the influence of the flow rate on film thickness on the catalyst surface is of major impact for diffusion limitation in future work, the influence of the flow rate will be tested. It is expected that a much higher flow rate may be beneficial for HMF yield at same conversion, as a faster diffusion of HMF out of the particles will reduce the possibility of side reactions. This can be attained by higher flow rates using a longer catalyst bed to achieve the same residence times.

## 3. Materials and Methods

### 3.1. Reactor Setup

**Figure 6:**
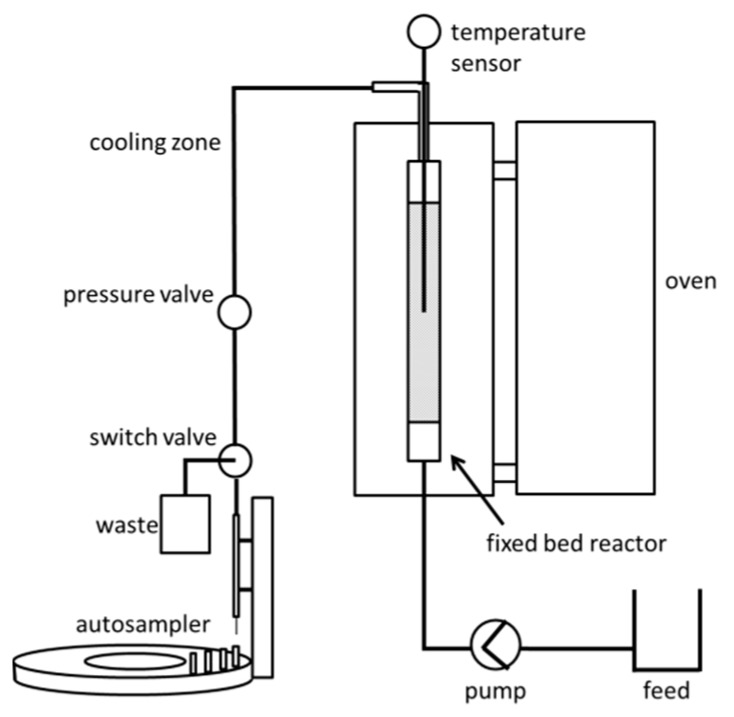
Fixed bed reactor setup.

The fixed bed reactor (Figure 6) embedded in the reactor oven (HTM-Reetz LK 500-40-400-1, Berlin, Germany) consists of a 15 cm × 0.95 cm (inner diameter) stainless steel tube (technical useful volume 6 mL), a catalyst bed of 1.7 g Lewatit K2420 (dry matter, measured by Ohaus MB45, Parsippani, NJ, USA), which is fixated by glass wool at the outlet and a frit at the inlet, as well as a Pt100 thermistor in the middle of the bed. The feed consisting of HFIP, fructose and water is pumped via an HPLC-pump (Knauer Smartline Pump 100, Berlin, Germany) through a 0.16 cm (inner diameter) PTFE pipe into the fixed bed reactor. At the reactor outlet, a 50 cm × 0.16 cm (inner diameter) PTFE pipe is connected, which acts as a cooling zone. To avoid vaporization of reaction solution, a pressure valve (Swagelok KCB Series, Solon, OH, USA) at the end of the cooling zone keeps the complete reactor setup at 20 bar pressure. Samples were taken with an autosampler (HiTec Zang, AutoSam 360, Herzogenrath, Germany) after the pressure valve.

The mean residence time in the reactor was calculated by the pulse method. A small amount of aqueous H_2_SO_4_ was pumped at different volume flow rates of pure water through the reactor whilst measuring the pH (Schott Instruments Lab860, Mainz, Germany with Mettler Toledo InLab Micro probe, Columbus, OH, USA) of the reactor outlet stream constantly.

### 3.2. HMF-Synthesis

Before starting the experiment, water was pumped through the heated reactor until the desired temperature (95–105 °C) was reached. Then, the feed was switched to the reaction solution consisting of 0.1 M fructose in the desired ratio of HFIP/water mixture (12.5–17.5 vol% water in HFIP). Three samples were taken after triple mean residence time. The standard deviations between all sets of samples were <1% in terms of conversion, yield of HMF and yield of by-products.

### 3.3. Materials

The following chemicals were used as received: Fructose (99.5% p.a. Carl Roth, Karlsruhe, Germany), HFIP (99%, Apollo Scientific, Bredbury, UK, 99% Flurochem, Hadfield, UK), water (Merck Milli-Q, Darmstadt, Germany), Lewatit K2420 (Lanxess, Cologne, Germany), levulinic acid (98%, Merck, Darmstadt, Germany), formic acid (98%, Carl Roth, Karlsruhe, Germany), HMF (98%, Sigma-Aldrich, St. Louis, MO, USA). Lewatit K2420 was washed with water until the permeate was colourless and was stored in water prior to use. HFIP was recycled by atmospheric evaporation distillation (bp. 58 °C) where no HFIP was detectable by HPLC in the remaining aqueous solution.

### 3.4. Methods

Quantitative analysis was performed with a Shimadzu HPLC (Kyoto, Japan) with Bio-Rad Animex HPX-87H column (Hercules, CA, USA) at 60 °C, 0.7 mL/min flow rate of 5 mM H_2_SO_4_ and refraction index detector and UV detector at 210 nm. Calibration was realised with external standards. All samples were diluted by a ratio of at least 1:10 with water (Merck Milli-Q) prior to analysis.

## 4. Conclusions

The flourous alcohol hexafluoroisopropanol shows superior performance for HMF synthesis from fructose catalysed by strong acidic ion exchange resin using a Lewatit K2420 in a continuous fixed bed reactor. Compared to previous batch experiments [14] the HMF yield is comparable (76% batch vs. 76% fixed-bed). Therefore, the transfer from batch process to continuous process was entirely successful.

Compared to the literature [5,6,7,9,10,11,12,15], the essential advantage is the use of the easy-to-separate solvent HFIP in a continuous fixed bed reactor system without using hard-to-separate high boiling solvents, homogeneous catalysts or phase modifiers. At the same time, yield and productivity of HMF are of a similar or even higher level.
